# Senescent fibroblast-derived Chemerin promotes squamous cell carcinoma migration

**DOI:** 10.18632/oncotarget.13446

**Published:** 2016-11-18

**Authors:** Vida Farsam, Abhijit Basu, Martina Gatzka, Nicolai Treiber, Lars A. Schneider, Medhanie A. Mulaw, Tanja Lucas, Stefan Kochanek, Reinhard Dummer, Mitchell P. Levesque, Meinhard Wlaschek, Karin Scharffetter-Kochanek

**Affiliations:** ^1^ Department of Dermatology and Allergic Diseases, University of Ulm, Germany; ^2^ Institute of Experimental Cancer Research, University of Ulm, Germany; ^3^ Department of Gene Therapy, University of Ulm, Germany; ^4^ Department of Dermatology, University Hospital Zurich, Switzerland

**Keywords:** cutaneous squamous cell carcinoma (cSCC), senescence-associated secretory phenotype (SASP), tumor migration, Chemerin, chemokine CC-motif receptor-like 2 (CCRL2)

## Abstract

Aging is associated with a rising incidence of cutaneous squamous cell carcinoma (cSCC), an aggressive skin cancer with the potential for local invasion and metastasis. Acquisition of a senescence-associated secretory phenotype (SASP) in dermal fibroblasts has been postulated to promote skin cancer progression in elderly individuals. The underlying molecular mechanisms are largely unexplored. We show that Chemerin, a previously unreported SASP factor released from senescent human dermal fibroblasts, promotes cSCC cell migration, a key feature driving tumor progression. Whereas the Chemerin abundance is downregulated in malignant cSCC cells, increased Chemerin transcripts and protein concentrations are detected in replicative senescent fibroblasts *in vitro* and in the fibroblast of skin sections from old donors, indicating that a Chemerin gradient is built up in the dermis of elderly. Using Transwell^®^ migration assays, we show that Chemerin enhances the chemotaxis of different cSCC cell lines. Notably, the Chemerin receptor CCRL2 is remarkably upregulated in cSCC cell lines and human patient biopsies. Silencing Chemerin in senescent fibroblasts or the CCRL2 and GPR1 receptors in the SCL-1 cSCC cell line abrogates the Chemerin-mediated chemotaxis. Chemerin triggers the MAPK cascade via JNK and ERK1 activation, whereby the inhibition impairs the SASP- or Chemerin-mediated cSCC cell migration.

Taken together, we uncover a key role for Chemerin, as a major factor in the secretome of senescent fibroblasts, promoting cSCC cell migration and possibly progression, relaying its signals through CCRL2 and GPR1 receptors with subsequent MAPK activation. These findings might have implications for targeted therapeutic interventions in elderly patients.

## INTRODUCTION

Cutaneous squamous cell carcinoma (cSCC) represents the second most common type of skin cancer worldwide with increasing incidence in elderly individuals [1]. cSCC develops through a multistep process, in which the accumulation of mutations and genetic alterations mainly drive the initiation step [2], while the cellular and molecular alterations of the surrounding microenvironment support the promotion and progression steps [3]. Exposure to ultraviolet (UV) radiation is the main cause for DNA damage and mutations in epidermal keratinocytes as well as in dermal fibroblasts underneath the epidermis [4, 5]. Mutations in the epidermal stem/progenitor cells affecting oncogenes like Ras or tumor suppressor genes such as p53 are frequently causal for cSCC initiation [2]. In addition, the DNA damage response pathways in dermal fibroblasts lead to the activation of p53 and p16INK4a and induction of cellular senescence. Senescent fibroblasts have been shown to accumulate over the life span in the skin of rodents, non-human primates and humans [6–8]. Senescent fibroblasts adopt a senescence-associated secretory phenotype (SASP), consisting of inflammatory cytokines, chemokines and matrix remodeling factors that - depending on the biological context - may contribute to tumor suppression or progression [7, 9]. Though the senescent human fibroblasts have been reported to accelerate epidermal tumorigenesis in nude mice, the underlying mediators are largely unknown [10]. It has been proposed that the activation of oncogenic Ras in aged murine skin causes excessive cellular senescence, epidermal stem cell dysfunction, enhanced inflammation, and immune cell skewing towards a T helper cell type 2, eventually resulting in epidermal dysplasia and cSCC progression [11].

Secreted chemokines are major components of the SASP [12], which play key roles in tumor cell motility, invasion and metastasis [13]. The involvement of chemokine/chemokine receptors in tumor metastasis was first dissected by Müller and colleagues. These authors uncovered the contribution of CXCL12/CXCR4 in breast cancer metastasis [14]. Subsequently, additional evidence substantiated the role of chemokines in tumor progression, particularly in tumor cell migration and invasion [13, 15, 16]. In this context, the CCL21/CCR7 axis has been proposed to be involved in the development of lymph node metastasis in a variety of different tumors [17–19]; and the CXCL6/CXCR6 axis has been shown to induce the progression of prostate [20] and breast cancers [21]

Of note, we found a remarkable upregulation of C-C chemokine receptor-like 2 (CCRL2) in cSCC primary tumors and cell lines as compared to the normal keratinocytes, their benign counterparts. CCRL2 has been earlier suggested to contribute to glioblastoma (GBM) cell migration and invasion [22] as well as to colorectal liver metastasis [23]. The known ligand for CCRL2 is the 18-kDa chemoattractant protein Chemerin [24]. Chemerin, also known as tazarotene-induced gene 2 (TIG2) [25] and retinoic acid receptor responder 2 (RARRES2), binds to two other independent receptors termed chemokine-like receptor 1 (CMKLR1 or ChemR23) [26] and G-protein coupled receptor 1 (GPR1) [27]. Overexpression of Chemerin was linked to enhanced tumor angiogenesis and poor clinical outcome in oral squamous cell carcinoma (OSCC) [28], and progression of esophageal squamous cell carcinoma (ESCC) [29, 30]. Increased serum Chemerin level was associated with cellular invasiveness in gastric cancer [31]. Here, we report markedly elevated Chemerin concentrations in the SASP of human senescent dermal fibroblasts, and uncover a previously unreported role for the Chemerin/CCRL2 axis in promoting MAPK-dependent cSCC migration.

Our findings may have substantial clinical impact for the development of therapeutic strategies to counteract the SASP-induced progression of cutaneous squamous cell carcinoma in elderly patients.

## RESULTS

### The secretome of senescent fibroblasts enhances migration and invasion of cutaneous squamous carcinoma cell lines *in vitro*

**Figure 1:**
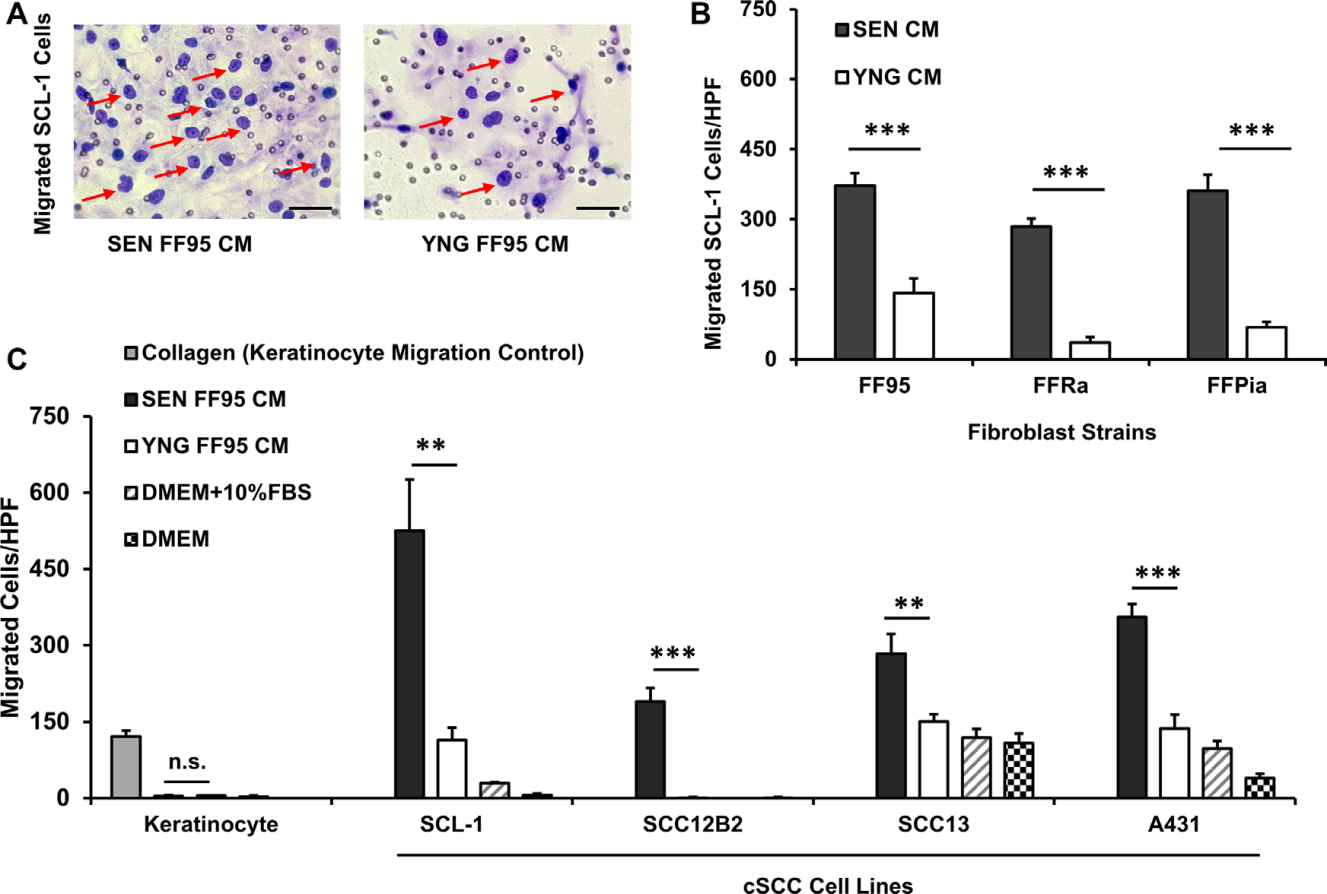
Secretome of senescent fibroblasts enhances the migration of squamous cell carcinoma lines Cell motility was evaluated using the Transwell^®^ chamber migration assay. The lower compartments of the chamber were loaded with conditioned medium (CM) of senescent (SEN) and corresponding young (YNG) fibroblasts and incubated for 14 h at 37°C. DMEM containing 10% fetal bovine serum (FBS) and collagen type I at a concentration of 50 μg/ml served as positive controls. DMEM served as a negative control. Cells stained purple by Diff-Quick staining kit and counted under light microscopy at ×100 magnification (**A**) Representative images (×200 magnification, scale bars = 50 μm) showing migrated SCL-1 cells (pointed with red arrows) at the downside of the membrane in response to CM from senescent vs. young fibroblasts. Quantifications of cell migration proved that (**B**) Senescent (SEN) conditioned media of human dermal fibroblast strains FF95, FFRa and FFPia enhanced the migration of SCL-1 tumor cells compared with young (YNG) counterparts. Data are shown as mean ± S.D for *n* = 3 replicates. ***p < 0.001 calculated by unpaired student *t*-test between SEN CM-treated and YNG CM- treated groups for each fibroblast strains. (**C**) Conditioned media of FF95 senescent fibroblasts increased the migration of all tested tumor cells (SCL-1, SCC-12B2, SCC-13 and A431) but had no effect on normal keratinocytes. Data are shown as mean ± S.D for *n* = 3 replicates; Graphs represent one of the three independent experiments; *p < 0.05, **p < 0.01 and ***p < 0.001 calculated by unpaired student *t*-test between SEN CM-treated and YNG CM- treated groups for each cell line; HPF = ×100 magnification. (Note that due to low standard deviations of some measurements, error bars are not visible for all data points.).

Enhanced cell motility constitutes an important step in cancer progression and metastasis [32]. As the incidence of cSCCs increases with age and senescent fibroblasts have been suggested to play a causal role in cSCC progression, we investigated the paracrine effects of senescent human dermal fibroblasts (HDFs) on cSCC cell migration and invasion. Conditioned medium (CM) was harvested from senescent and young (early passage) fibroblasts to assess its chemotactic activity on migration of normal human keratinocytes and different cSCC cell lines (SCL-1, SCC-13, SCC-12B2 and A431 cells) in a Transwell^®^ chamber migration assay. As depicted in Figure 1A and 1B, increased cSCC cell migration was observed in response to senescent CM of FF95 fibroblast strain compared to young CM. Normal keratinocytes did not migrate when exposed to senescent or young fibroblast CM, as opposed to the strong chemotaxis in response to collagen (positive control) [33]. These results were reproduced using CM of all three independent replicative senescent HDF strains (FF95, FFRa and FFPia), confirming that the secretome of all tested senescent HDFs consistently enhanced the migration of SCL-1 tumor cells (Figure 1C). Although to a different extent, a robust migratory response of different cSCC cell lines was induced by CM of senescent fibroblasts of different strains (Figure 1C), these data indicate that the observed enhanced migratory response is similar for all tested cSCC cell lines and senescent fibroblast strains and most likely constitutes a general mechanism of SASP-induced migration. Interestingly, CM of primary stromal fibroblasts derived from cSCC patients at old age displayed similar migratory-promoting effects on SCL-1 cells (Supplementary Figure S1).

Moreover, we observed an increased cell invasion through a reconstituted basement membrane (Matrigel^®^) in response to conditioned medium of senescent fibroblasts (FF95 CM) in SCL-1 cancer cells *in vitro* (Supplementary Figure S2). This phenotype has been previously reported to be mediated through the secretion of active MMP-2 by senescent cancer-associated fibroblasts [34].

### The chemoattractant Chemerin is upregulated in senescent fibroblasts

Earlier we attempted to define the secretome of senescent fibroblasts using an antibody array, mainly confirming the previously published SASP factors [6, 35, 36]. Even though these SASP factors, such as CCL5/RANTES [37, 38], were able to significantly stimulate cSCC cell migration (Supplementary Figure S3), they were produced at even higher levels by SCC cells themselves in an autocrine manner, as have been previously reported [39, 40]. Therefore, any significant paracrine contribution from senescent dermal fibroblasts was ruled out.

In a complementary attempt to identify novel SASP factors, we performed PCR array analysis of the chemokine receptors in cSCC cells (Supplementary Figures S4 and S5). Of note, we found a remarkable upregulation of CCRL2 receptor in all tested cSCC cell lines, a chemokine receptor processing high affinity for Chemerin, the ligand which had not been identified with the conventional screening strategies.

**Figure 2:**
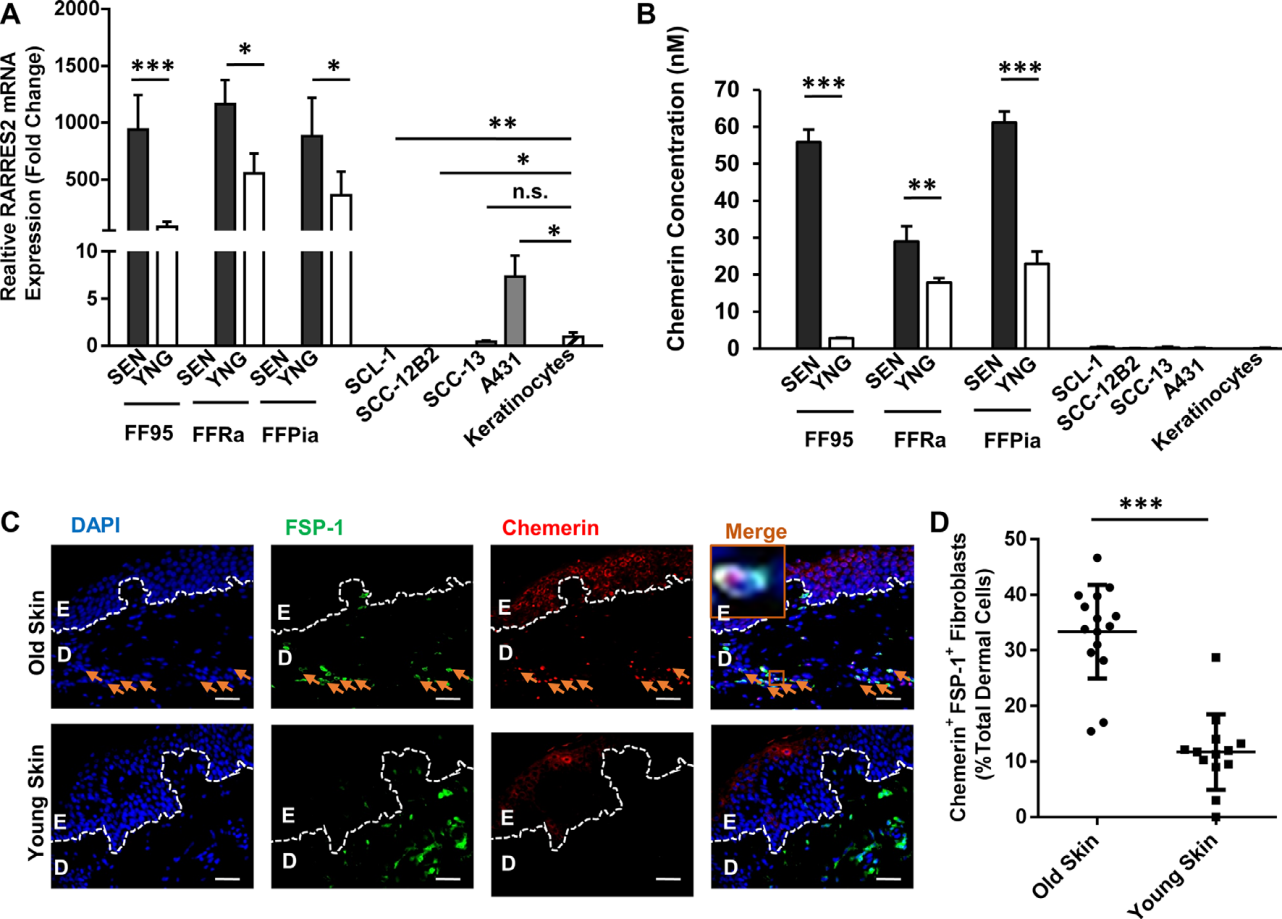
Chemerin is an upregulated SASP factor in human dermal fibroblasts (**A**) Graph demonstrating the relative RARRES2 (Chemerin gene) mRNA expression in senescent (SEN) vs. young (YNG) fibroblast of different strains (FF95, FFRa and FFPia) as defined by qRT-PCR. Data are normalized to the expression level of RARRES2 in keratinocytes, confirming that the senescent fibroblasts display the highest, and the cSCC cell lines (SCL-1, SCC12-B2, SCC-13) display the lowest RARRES2 transcripts, respectively. Date are shown as mean ± S.D for one of three independent experiments of biological replicates (*n* = 3); *p < 0.05, **p < 0.01 and ***p < 0.001 calculated by Bonferroni post hoc test after ANOVA. (**B**) Chemerin secretion was analyzed in the above mentioned cells (normalized to 5 × 10^6^ cells/ml) using ELISA. Data are shown as mean ± S.E.M for three independent experiments; *p < 0.05, **p < 0.01 and ***p < 0.001 calculated by Bonferroni post hoc test after ANOVA. (Note that due to low standard deviations of some measurements, error bars are not visible for all data points.) (**C**) Representative photomicrographs of paraffin-embedded human skin sections co-immunostained with anti-FSP-1 antibody in green and anti-Chemerin antibody in red, depicting higher abundance of Chemerin in skin dermal fibroblasts of aged (70-year old), compared to young (23-year old) donors. Nuclei were DAPI-counterstained (blue). Appropriate isotype controls were used to determine the background. Scale bars = 50 μm at ×400 magnification; Dashed lines delineate epidermis (E) from dermis (D). Orange arrows point to the Chemerin-positive fibroblasts. Orange boxes depict the magnified area. (**D**) Graph representing the quantification of Chemerin-positive fibroblasts (shown by FSP-1 marker) in the skin dermis of old healthy individuals (76 ± 10 year, *n* = 15 donors) and young (21 ± 8 year, *n* = 13 donors) calculated from minimum 5 technical replicates. ***p < 0.001 by two-tailed student *t*-test.

Interestingly, the RARRES2 transcripts encoding the Chemerin protein were increased in all tested senescent fibroblast strains compared to young fibroblasts (Figure 2A). By contrast, with the exception of the A431 cell line, cSCC cells displayed significantly lower RARRES2 mRNA transcripts with a strong downregulation of Chemerin expression as compared to normal cells (keratinocytes) and fibroblasts (Figure 2A).

Enzyme-linked immunosorbent assay (ELISA) revealed increased levels of secreted Chemerin in CM of senescent fibroblasts compared with CM of young fibroblasts with a very low expression in keratinocytes to almost undetectable in cSCC cells (Figure 2B). These data were further confirmed by immunostaining (Figure 2C and 2D), showing significantly higher number of Chemerin-positive fibroblasts (33.35 % ± 8.22 of total dermal cells) in skin biopsies derived from aged healthy human individuals (age 76 ± 10 years, *n* = 15 healthy donors) as compared to young humans (11.82 % ± 6.82 total dermal cells, age 21 ± 8 years, *n* = 13 healthy donors). Of note, immunostaining of skin biopsies derived from cSCC patients (Supplementary Figure S6) revealed that a large number of Chemerin-releasing FSP-1^+^ fibroblasts (30.83 % ± 1.89 of total stromal cells) were present in the cSCC tumor stroma of older patients (age 80 ± 5 years, *n* = 5 cSCC patients) as compared to younger cSCC patients (16.04 ± 3.2 total stromal cells, age 54 ± 5 years, *n* = 5 cSCC patients).

### Chemerin significantly increases the migration of cSCC cell lines *in vitro*

**Figure 3:**
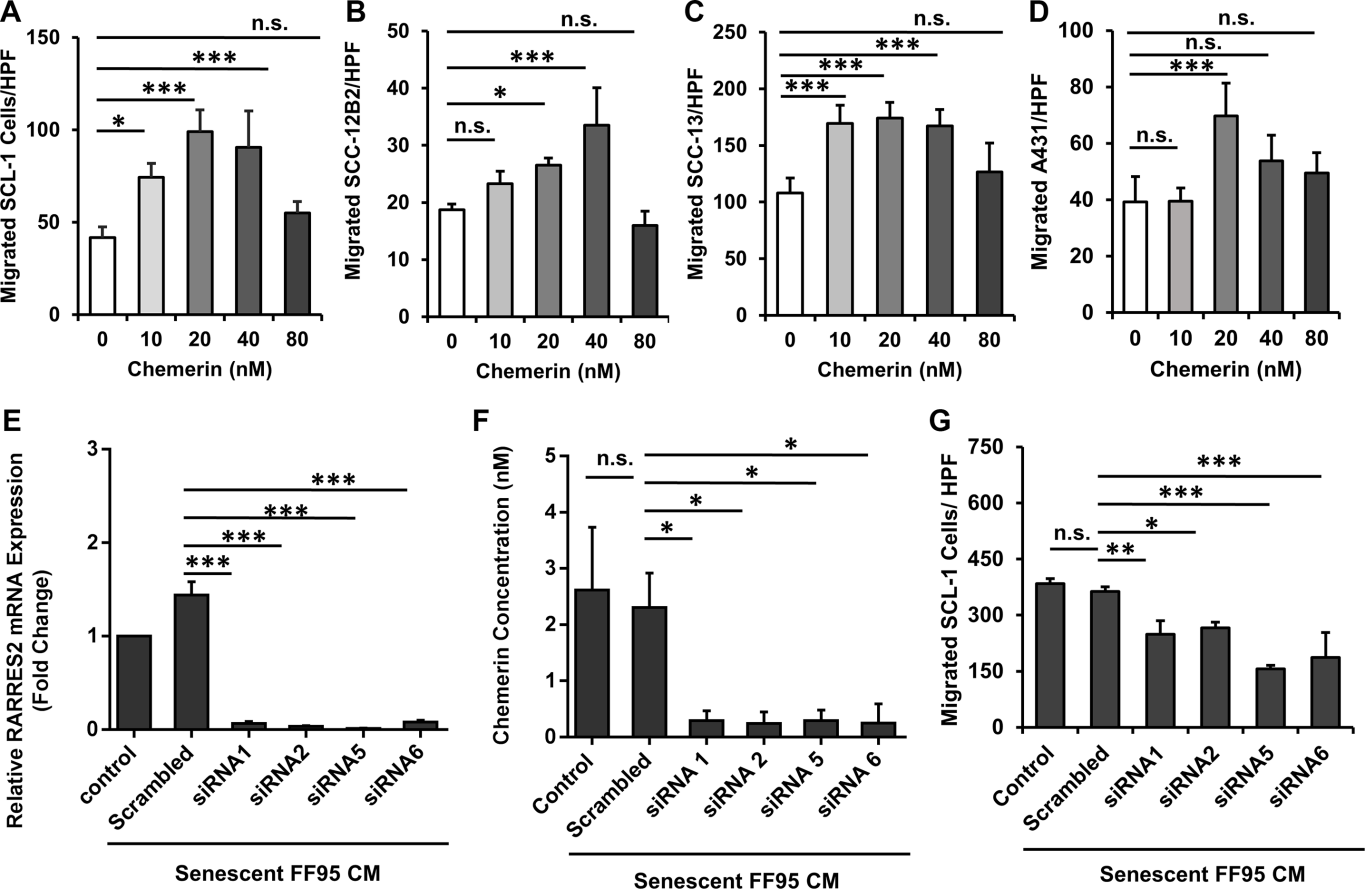
Chemerin stimulates migration of squamous carcinoma cell lines Graphs demonstrating the bell-shape curves of chemotactic response to the increased concentrations of Chemerin in (**A**) SCL-1, (**B**) SCC-12B2, (**C**) SCC-13 and (**D**) A431 cells using the Transwell^®^ chamber migration assay. Date are shown as mean ± S.D for one of three independent experiments with *n* = 4 replicate wells; n.s. = non-significant, *p < 0.05, **p < 0.01 and ***p < 0.001 in comparison to random migration control with no Chemerin treatment calculated by Bonferroni post hoc test after ANOVA. The role of Chemerin in mediating cSCC cell migration was confirmed by silencing the Chemerin gene (RARRES2) in senescent fibroblasts and assessing their ability to induce SCL-1 cell migration (**E**) Transcript level of RARRES2 was quantified by qRT-PCR in senescent fibroblasts 42 h post treatment with siRNAs, confirming the successful gene silencing compared to scrambled control. Data are shown as mean ± S.E.M from three independent experiments. ***p < 0.001 calculated by Bonferroni post hoc test after ANOVA. (**F**) Chemerin protein level (normalized to 5 × 10^6^ cells/ml) was measured 48 hours after treatment with siRNAs or scrambled control. Data are shown as mean ± S.E.M from four independent experiments; *p < 0.05 calculated by Bonferroni post hoc test after ANOVA. (**G**) Conditioned media derived from Chemerin-silenced fibroblasts (siRNA 1, 2, 5 and 6) were used to induce the migration of SCL-1 cells using the Transwell^®^ chamber migration assay. Conditioned media derived from mock-treated fibroblasts (Scrambled) and senescent fibroblasts with no treatment (control) were used as controls. Shown is one representative of four independent experiments, each with triplicate samples. *p < 0.05, **p < 0.01 and ***p < 0.001 calculated by Bonferroni post hoc test after ANOVA.

To determine whether cSCC cells display a migratory response to the gradients of recombinant human (rh) Chemerin, DMEM containing different concentrations of rh Chemerin was placed in the bottom compartment of the Transwell^®^ chambers with cSCC cell lines seeded on top of the perforated membrane in the upper compartment. Chemerin induced a significant increase in the migration of all assessed cSCC cell lines compared with the untreated control (the number of cells migrating spontaneously in the absence of a chemotactic Chemerin gradient). The increase in the migratory response occurred concentration-dependently, demonstrating a typical bell-shaped response curve with maximal numbers of migrated cells at concentrations between 20 to 40 nM Chemerin (Figure 3A–3D). Based on these results the concentration of 40 nM Chemerin was used for subsequent experiments. Of note, we could show that the migratory response of cSCC cells to rh Chemerin alone occurred at a physiological Chemerin concentration of 20–40 nM (< 150 cells/HPF), while the CM of senescent fibroblasts induced an even stronger migratory response of cSCC cells (> 300 cells/HPF), suggesting that other SASP factors in addition to Chemerin are contributing. In fact, we identified the SASP factor RANTES/CCL5 to induce cSCC cell migration (Supplementary Figure S3).

Of note, Chemerin does not induce cSCC cell proliferation *in vitro* as shown by MTT test and BrdU incorporation assays (Supplementary Figures S7A–S7E). These observations provide convincing evidence in favor of the hypothesis that migration and proliferation are mutually exclusive in cancer cells [41, 42].

### Chemerin depletion diminishes the potential of senescent fibroblast-derived SASP to induce cSCC cell migration

To further assess whether Chemerin contributes to the SASP-induced migratory response of cSCC cells, Chemerin was depleted from senescent fibroblasts using Chemerin-specific silencing RNAs. Two days after transfection, RARRES2 transcripts and Chemerin secretion dramatically decreased to less than 5% of the mock-treated fibroblasts as shown with qRT-PCR and ELISA (Figure 3E and 3F), respectively. Of note, CM derived from Chemerin-silenced senescent fibroblasts showed a significant reduction (over 30%) in stimulating the migratory response of SCL-1 cells (Figure 3G). This finding was confirmed with four independent silencing RNAs, indicating that Chemerin is one of the major SAPS factors of senescent dermal fibroblasts, which induces cSCC cell migration.

### The Chemerin receptor CCRL2 is upregulated in cSCC cells

As mentioned above, the higher level of CCRL2 receptor expression was detected on cSCC cells compared with normal cells using the PCR array analysis.

**Figure 4:**
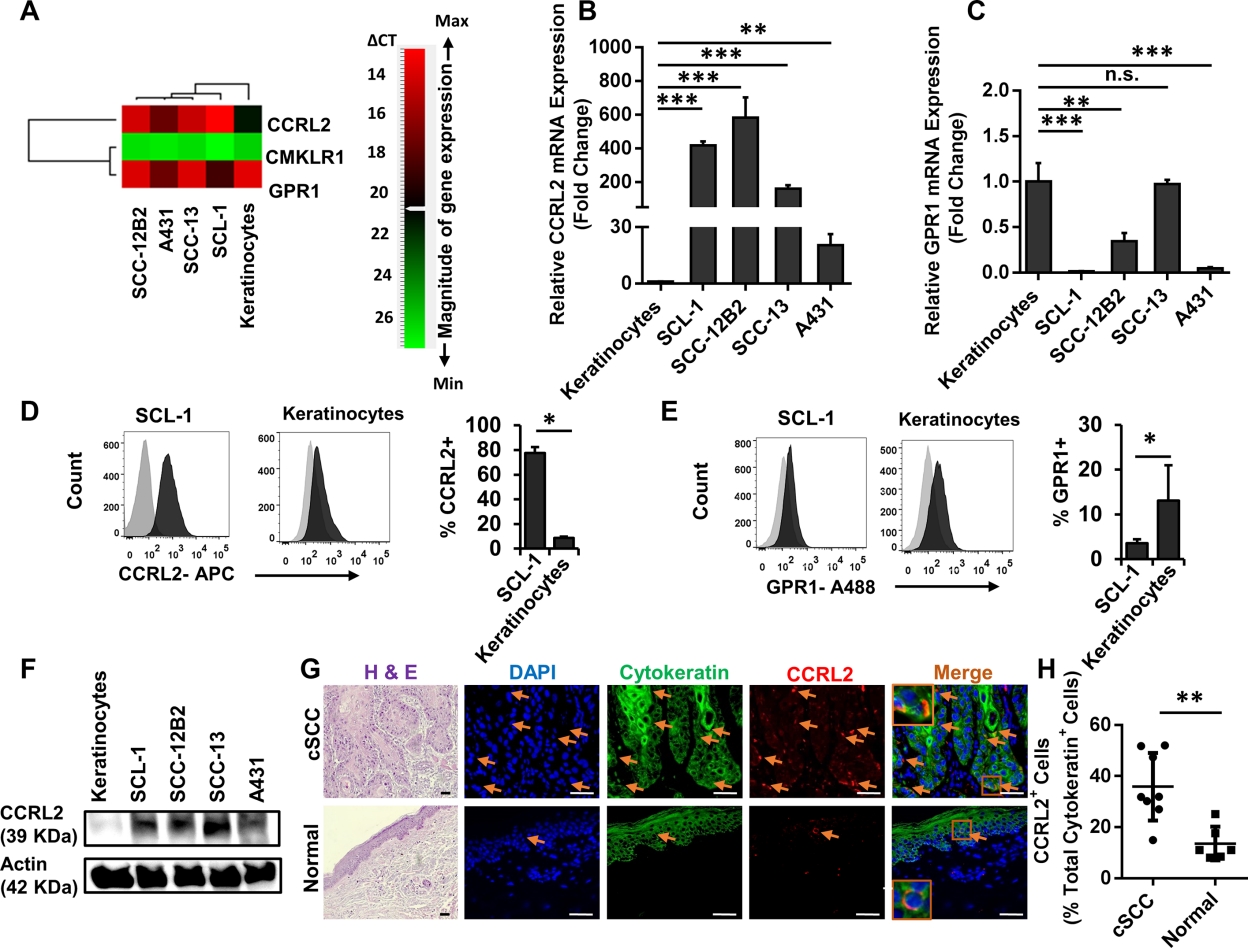
CCRL2 is the upregulated Chemerin receptor in cutaneous carcinoma cell lines and human squamous cell carcinoma (**A**) Heat map representing the gene expression profile of Chemerin receptors CCRL2, GPR1 and CMKLR1. Relative gene expression was calculated from all three independent experiments normalized to the level of Actin housekeeping gene expression, and relative to the mean expression in normal keratinocytes. Genes were rank-ordered by Pearson correlation and depicted by a pseudo color scale based on qRT-PCR delta-CT values with green and red demonstrating low and high mRNA abundance, respectively. CMKLR1 had very low to no mRNA transcripts; CCRL2 was highly upregulated in cSCC cells; and GPR1 was either downregulated or not changed in cSCC cells compared to keratinocytes. (**B**–**C**) Graphs depict the relative fold changes of CCRL2 and GPR1 mRNA expression in cSCC lines compared with normal keratinocytes as defined by qRT-PCR. Shown is one representative of three independent experiments, each with triplicate samples. *p < 0.05, **p < 0.01 and ***p < 0.001 calculated by Bonferroni post hoc test after ANOVA. (Note that due to low standard deviations of some measurements, error bars are not visible for all data points.) (**D**–**E**) Cell surface expressions of CCRL2, GPR1 and CMKLR1 were assessed by flow cytometry analysis in SCL-1 cells and normal keratinocytes. Displayed are the representative fluorescence histograms depicting the relative fluorescence intensity of cells stained with (D) APC-conjugated anti-CCRL2 and (E) A488-conjugated anti-GPR1 antibodies (black histograms) compared with isotype control antibodies conjugated with corresponding fluorochromes (gray histograms). The expression of CMKLR1 was not detectable (not shown). Bar charts represent one of three independent experiments demonstrating the mean percentage ± S.D. for (d) CCRL2-positive and (E) GPR1-positive SCL-1 cells vs. keratinocytes. *p < 0.05, **p < 0.01 and ***p < 0.001 calculated by two-tailed student *t*-test (*n* = 8 biological replicates). (**F**) Representative Western bot analysis of cSCC and keratinocyte cell lysates, confirming the elevated protein level of CCRL2 in cSCC cells compared with normal keratinocytes. (**G**) Representative photomicrographs of skin biopsies derived from patients suffering from invasive cSCC vs. normal healthy controls stained for Hematoxylin and Eosin (H & E), CCRL2 in red and cytokeratin in green. Nuclear was stained with DAPI (blue). Arrows show the CCRL2-positive cells. Appropriate isotype controls were used to determine the background. H&E images were taken at ×200 magnification and immunofluorescence at ×400 magnification; Scale bars = 50 μm. Orange boxes depict the magnified area. (**H**) Graph representing the quantification of CCRL2-positive epidermal-derived cells in the skin specimens of patients suffering from cSCC (*n* = 8) vs. normal healthy individuals (*n* = 6). **p < 0.01 by two-tailed student *t*-test.

We further employed qRT-PCR, Western blot and flow cytometry analysis to evaluate the expression of CCRL2 and other Chemerin receptors including CMKLR1 and GPR1 in cSCC cell lines and normal keratinocytes (Figure 4). Notably, CCRL2 mRNA was significantly increased in all cSCC cells (> 25-fold) compared to normal keratinocytes (Figure 6A, 6B and Supplementary Figures S4 and S5). GPR1 mRNA was also detectable in cSCC cell lines, although the transcripts were substantially less abundant in comparison with normal keratinocytes (Figure 4A and 4C).

Transcripts of CMKLR1 were neither detected in cSCC cell lines, nor in keratinocytes (Figure 4A).

Using flow cytometry analysis, the cell surface expression of CCRL2, GPR1 and CMKLR1 was analyzed, revealing high expression levels of CCRL2 (77.7% ± 4.6) and low expression of GPR1 (3.55% ± 0.87) on SCL-1 cells in comparison to a significantly lower level of CCRL2 (8.7% ± 1.1) and higher GPR-1 (13.1% ± 7.92) on keratinocytes (Figure 4D and 4E). Neither SCL-1 cells nor keratinocytes expressed CMKLR1 at detectable levels (data not shown).

Western blot analysis confirmed the upregulation of CCRL2 at the protein level; Consistent with the FACS analysis and qRT-PCR data, SCL-1, SCC-12B2, SCC-13 and A431 strongly expressed CCRL2 compared with normal keratinocytes (Figure 4F).

To confirm the clinical significance of CCRL2 expression, primary tumors from cSCC patients (*n* = 8) were subjected to immunofluorescence staining. CCRL2 staining was mainly observed in the invading edges of cSCC cells. By contrast, in normal skin from healthy individuals, epidermal keratinocytes displayed no or very low CCRL2 expression (Figure 4G).

Hence, CCRL2 constitutes the most abundant Chemerin receptor in cSCC cells, suggesting that it may favor the enhanced migration of cSCC tumor cells to invade the Chemerin-rich dermis.

### Role of CCRL2 and GPR1 in Chemerin-mediated cSCC cell migration

**Figure 5:**
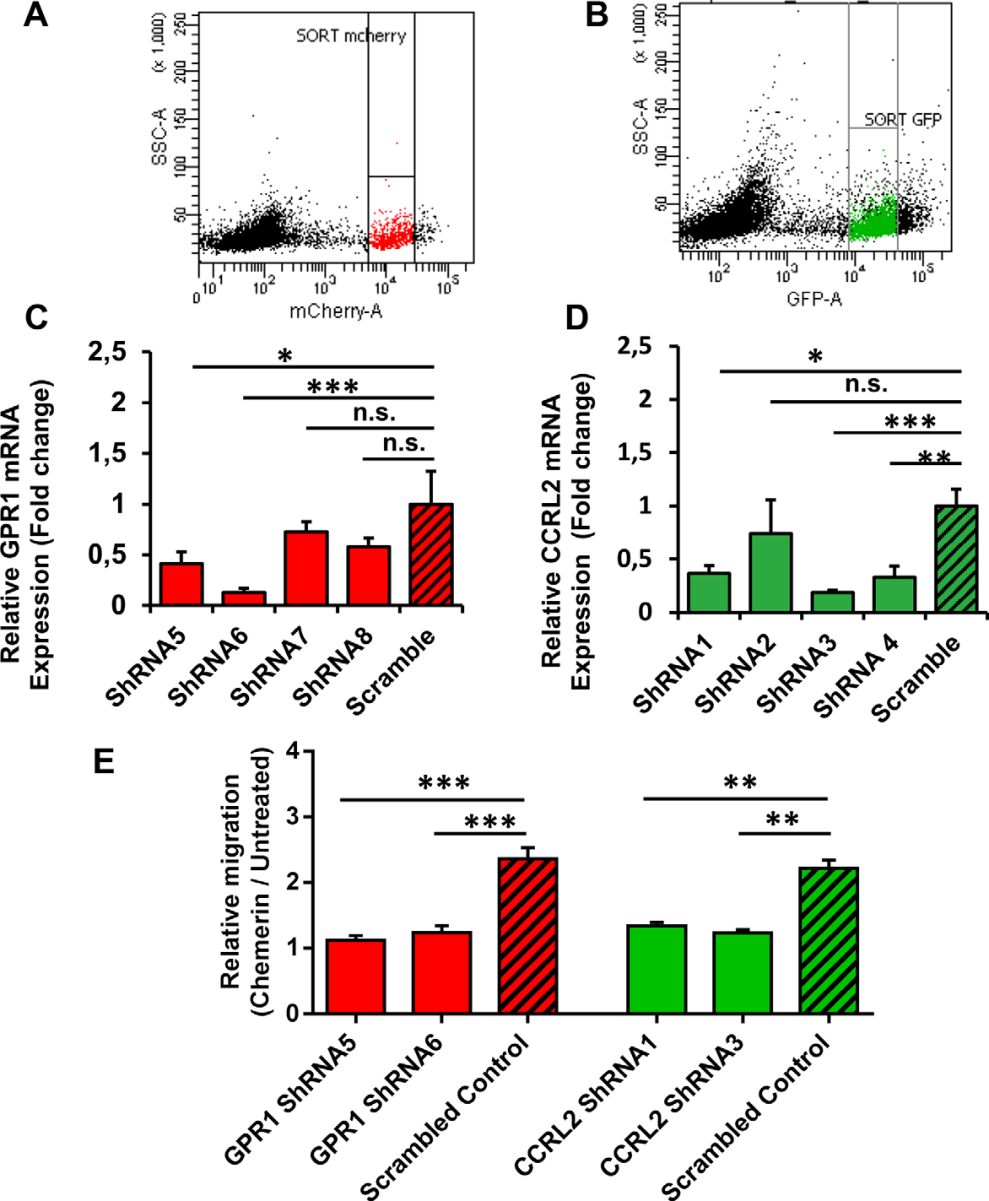
CCRL2 and GPR1 receptors are required for Chemerin-stimulated squamous cell carcinoma migration In order to study the role of Chemerin receptors in mediating cSCC cell migration, SCL-1 cells were transduced with lentivirus encoding shRNAs against GPR1 and CCRL2. The GPR1 shRNA-expressing lentiviral vector and the CCRL2 shRNA-expressing lentiviral vector contained mCherry and eGFP proteins, respectively, which enabled us to isolate positively-transduced cells using fluorescence-activated cell sorting (FACS). (**A**–**B**) Representative FACS dot plots showing the final gating strategies to sort mCherry- and eGFP-expressing cells. (**C**–**D**) The efficiency of CCRL2 and GPR1 knockdown in SCL-1 cells was validated by qRT-PCR. Data are presented as bar charts, representing the mean expression fold changes relative to scrambled controls ± S.D. *p < 0.05, **p < 0.01 and ***p < 0.001 calculated by ANOVA (*n* = 3). (**E**) Graph indicating that CCRL2 and GPR1 silencing suppresses Chemerin-mediated SCL-1 cell migration. The relative migration (^Chemerin^/_Untreated_) was calculated in each group as the ratio of cells migrated towards the Chemerin gradient (40 nM) to the cells migrated randomly in the negative control (serum-free DMEM). Data are presented as Mean ± S.E.M. for 3 independent experiments; *p < 0.05, **p < 0.01 and ***p < 0.001 calculated by Bonferroni post hoc test after ANOVA.

Enhanced CCRL2 expression on cSCC cells indicates that this receptor might play a role in the migratory response to Chemerin. We therefore assessed whether the knockdown of CCRL2 expression using lentivirus-based short hairpin RNAs (shRNAs) could suppress the cSCC cell migration. Likewise, the GPR1 receptor was also lentivirally-silenced from SCL-1 cells. Successful transcriptional silencing of GPR1 and CCRL2 (> 50%) in sorted populations of mCherry-positive (GPR1) or eGFP-positive (CCRL2) SCL-1 cells (Figure 5A–5B) was verified using qRT-PCR (Figure 5C–5D).

The GPR1-silenced and CCRL2-silenced cells were subjected to Transwell^®^ chamber migration assays. Relative cell migration (^Chemerin^/_Untreated_) was calculated in each group as the ratio of cells migrated towards a Chemerin gradient (40 nM) to the cells migrated randomly in the negative control (untreated cells in serum-free DMEM). The results showed that the directed cell migration in response to rh Chemerin was reduced to a similar level as random migration in GPR1-knockdown or CCRL2-knockdown cells (^Chemerin^/_Untreated_ ~ 1) as compared to the scrambled-transfected cells (^Chemerin^/_Untreated_ > 2). In other words, the shRNA-mediated silencing of either CCRL2 or GPR1 blocked the Chemerin-induced migration by > 50% in SCL-1 cells in comparison to scrambled control (Figure 5E). Consistent results were achieved with both independent shRNAs targeting CCRL2 and GPR1 (Figure 5E).

These results support the notion that CCRL2 and GPR1 constitute the responsible receptors for Chemerin-induced cSCC cell migration. This is consistent with our observation that keratinocytes, despite expressing high levels of GPR1, are not responsive to Chemerin, probably due to the very low CCRL2 expression (data not shown).

### Activation of MAPK signaling pathway in response to Chemerin

**Figure 6:**
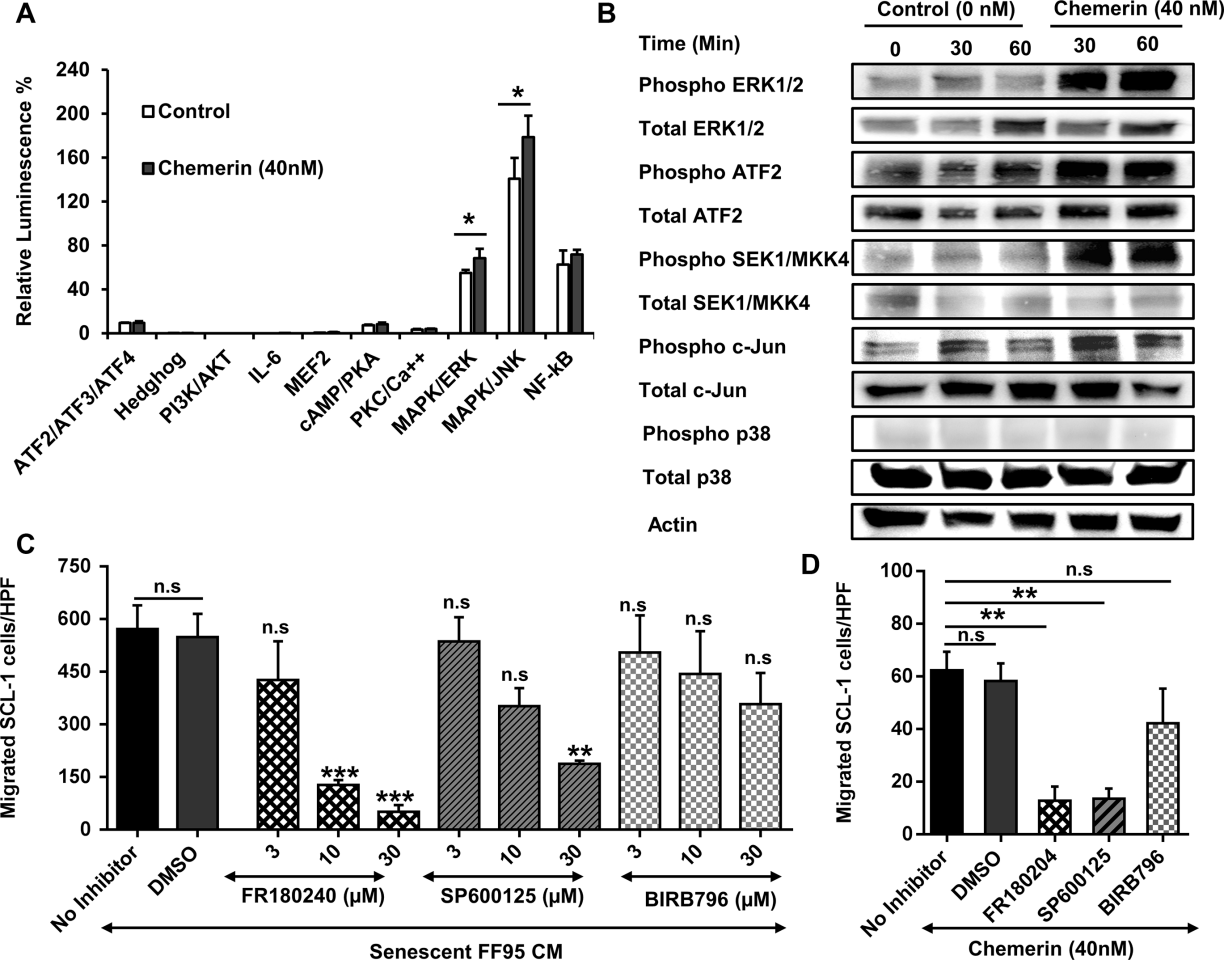
Role of MAPK signaling pathway in the regulation of Chemerin-mediated cell migration (**A**) In order to define the signaling pathway activated by Chemerin, we used a G-protein couple receptor (GPCR) Cignal Finder^®^ Reporter Array consisting of inducible transcription factor response constructs encoding the firefly luciferase reporter gene. SCL-1 cells were transfected with each reporter constructs and further treated with DMEM containing 40 nM rh Chemerin or no Chemerin (control) for 1 hour. The dual-luciferase assay was developed and the results were expressed as the percentage of relative luminescence signal according to the manufacturer's instructions. Results highlighted that Chemerin activates JNK and ERK1/2 MAPK signaling pathway. Shown is a representative of three independent experiments presented as Mean ± S.D (*n* =3). *p < 0.05 calculated by unpaired student *t*-test. (Note that due to low standard deviations of some measurements, error bars are not visible for all data points.) (**B**) To confirm the Cignal Finder^®^ Reporter Array data, SCL-1 cells were cultured in the presence and absence of 40 nM rh Chemerin for 30 and 60 minutes and protein lysates were analyzed by Western blot for phosphorylated and total amounts of MAPK key proteins. (**C**) We investigated whether inhibition of MAPK pathway dampens the migration of SCL-1 cells stimulated by Chemerin-rich conditioned media of senescent fibroblasts. The graph represents the concentration-dependent inhibition of SCL-1 cell migration in response to senescent fibroblast CM by SP600125 (JNK inhibitor), FR180204 (ERK1/2 inhibitor) and BIRB796 (P38 inhibitor). DMSO served as a vehicle control. Data are presented as Mean ± S.E.M. for 3 independent experiments; *p < 0.05, **p < 0.01 and ***p < 0.001 in comparison to DMSO control calculated by Bonferroni post hoc test after ANOVA; HPF= ×100 magnification (**D**) Bar graph showing the effect of MAPK inhibitors (10 μM) on Chemerin-mediated SCL-1 cell migration. Data are presented as mean ± S.E.M. for 3 independent experiments; ***p < 0.001 calculated by Bonferroni post hoc test after ANOVA. HPF= ×100 magnification.

To further elucidate the molecular mechanism underlying Chemerin-induced cSCC cell migration, we explored signaling pathways downstream of the Chemerin receptors CCRL2 and GPR1, both belonging to the family of G-protein coupled receptors (GPCRs). We employed a GPCR Cignal^®^ Finder Array, and transfected SCL-1 cells with luciferase reporter DNA constructs containing transcription factor elements (TFEs), and assessed the reporter activities in response to rh Chemerin stimulation. Rh Chemerin enhanced Mitogen-activated protein kinase (MAPK) activities of both c-Jun N-terminal kinases (JNK) and extracellular-signal-regulated kinase (ERK) signaling pathways in SCL-1 cells after an incubation period of 1 h (Figure 6A). The activation of JNK and ERK persisted for up to 24 hours (data not shown).

Activation of MAPK pathways in response to rh Chemerin was further confirmed at the protein level by Western blot analysis. Increased phosphorylated forms of ATF2, SEK1, c-Jun and ERK1/2, were detected in rh Chemerin-treated SCL-1 cells compared with untreated cells (Figure 6B).

### Inhibition of MAPK pathway abrogates cSCC cell migration

Next we addressed the question whether the inhibition of the MAPK pathway can suppress Chemerin- or SASP-mediated migration of cSCC tumor cells. Notably, the ERK1/2 inhibitor FR180204 [43], and the JNK inhibitor SP600125 [44], in a concentration-dependent manner, mitigated the ability of senescent CM to induce SCL-1 cell migration. The SASP-mediated SCL-1 cell migration was almost completely abolished following treatment with 30 μM FR180204 and to a lesser extent with SP600125 (Figure 6C). BIRB796, a specific p38 inhibitor [45, 46], did not significantly decrease the SASP-induced migration of SCL-1 cells.

A concentration of 10 μM FR180204 or SP600125 but not BIRB796 blocked the rh Chemerin-induced SCL-1 cell migration (> 70%) in comparison to control (Figure 6D), suggesting that Chemerin regulates the cSCC cell migration through ERK1/2 and JNK- but independently of the p38 MAPK pathway.

The used concentrations were non-toxic as shown by MTT assays (Supplementary Figure S7F).

## DISCUSSION

Accumulating evidence suggests that the senescence-associated secretory phenotype (SASP) is one of the major causal links between the age-associated alterations in tissue microenvironment and the rising incidence of cancer [9, 11, 47]. It has been recently shown that the SASP from senescent stromal fibroblasts inhibits the RhoA/ROCK/myosin-dependent cell contractility leading to aggressive cell motility in human breast cancer cells [48]. However, the exact key SASP factors and the mechanisms involved in cSCC cell migration have not been fully elucidated. Here we showed that Chemerin, released by senescent dermal fibroblasts, exerts a strong chemotactic activity in cSCC cells and thus contributes to enhanced cSCC cell migration via activation of MAPK pathway. These findings were corroborated by our observation that the putative Chemerin receptor, CCRL2, was remarkably upregulated in cSCC tumor cells, which, together with the GPR1 receptor, mediated the response to Chemerin.

In general, Chemerin is known to be involved in regulating adipogenesis and lipid metabolism [49], inflammation and leukocyte trafficking [50], and endothelial angiogenesis and MMP production [51]. Dysregulated expression of Chemerin has been correlated with tumor progression in glioma [52], squamous cell carcinoma of oral tongue [28], esophageal cancer [30] and with tumor suppression in melanoma [53]. However, the biological effects of differential expression of Chemerin in tumor and stromal cells in the context of tumor migration have not been sufficiently explored. In the present study, based on the following findings, we conclude that the Chemerin/CCRL2/GPR1 axis is induced by the paracrine action of senescent fibroblasts, causing enhanced cSCC migration and most likely progression.

First, a comprehensive transcriptional analysis of the chemokine receptor expression in cSCC cell lines revealed a remarkable upregulation of CCRL2 in cSCC cell lines compared to normal keratinocytes (Supplementary Figures S4 and S5), a finding which was further confirmed with the immunostaining of patient tumor biopsies. The elevated CCRL2 expression in other tumors including malignant breast cancer [54], high grade glioblastoma [22] and cervical carcinoma [55] has been previously postulated to be associated with cell migration, invasion and poor prognosis, irrespective of whether its biological ligands were present or not. Thus, the high abundance of CCRL2 receptor on cSCC cell surface implicates a crucial role in tumor progression. We assumed that the CCRL2 upregulation confers a migratory advantage for the epidermal cSCC tumor cells, particularly at the invasive front of the epidermis-dermis junction, facilitating their migration towards the dermis, where a gradient of its high-affinity ligand, Chemerin, is established by senescent fibroblasts. Consistent with this notion, we detected significantly high Chemerin concentrations in senescent dermal fibroblasts *in vitro,* as well as in the dermal fibroblasts of old healthy individuals and in the stromal fibroblasts of old cSCC patients *in situ*. This expression pattern was significantly different in the young healthy skin, exhibiting lower Chemerin abundance in the dermis vs. epidermis, as previously demonstrated by Banas *et al.* [56]. Interestingly, in line with a previous publication [57] we found that Chemerin production was significantly decreased in cSCC cells compared to normal keratinocytes.

Second, as to the functional consequences of senescence-associated Chemerin upregulation in old skin, we showed that rh Chemerin increased the directed cSCC cell migration; and silencing of Chemerin in senescent fibroblasts significantly reduced their potential to induce cSCC cell migration. To our knowledge, this is the first report on the role of Chemerin in cSCC cell migration, although Chemerin has been previously reported to promote gastric cancer and esophageal cancer cell invasion via induction of VEGF, MMPs and IL-6 [30, 31].

Third, we showed that, in the absence of CMKLR1, both the CCRL2 and GPR1 receptors are required for Chemerin-mediated cSCC cell migration, as silencing of either receptors suppressed the migratory response of cSCC cells to a Chemerin gradient. In agreement with this finding, previous reports have demonstrated that the CCRL2 upregulation through an undefined ligand contributes to glioblastoma cell migration [22] and colorectal metastasis [23]. By contrast, CCRL2 overexpression was reported to inhibit CCL2-induced phosphorylation of p38 MAPK leading to decreased chemotaxis and invasion of human breast cancer cells [58]. Thus, the involvement of CCRL2 in Chemerin-induced cell migration, as a key step in tumor progression, is still confounding as to different tumor entities. This notion is based on the finding that CCRL2 possesses an altered amino acid sequence (QRYLVFL in huCCRL2 and QRYRVSF in mCCRL2 instead of the conserved DRYLAIV motif) in the second intracellular loop which is essential for signal transduction [24]. In this context, our data on GPR1/CCRL2/Chemerin-induced cell migration are novel and would fit to the current model of CCRL2 action, proposed by Zabel and colleagues [24]. The model suggests that Chemerin binds to CCRL2 receptors via its amino-terminal domain, which in consequence increases the abundance of cell surface-bound Chemerin and its local concentration. Chemerin then, in a juxtacrine fashion, reacts with other Chemerin receptors (e.g. CMKLR1 or GPR1) via its free carboxyl-terminal residues. More detailed analysis would, however, be required to further delineate the exact mechanism whereby Chemerin interacts with GPR1 and/or CCRL2 to stimulate cSCC migration.

Fourth, we showed that Chemerin triggers the mitogen-activated protein kinase (MAPK) signaling pathway in CCRL2^high^ GPR1^low^ SCL-1 cells which express no detectable CMKLR1 proteins. Previously, Chemerin was shown to activate the p38 and ERK1/2 MAPK pathways in gastric cancer cells [31], and PI3K, Akt, and p38 in CMKLR1-expressing macrophages [59]. In the current study, using the GPCR Cignal^®^ Finder array and Western blot analysis, the ERK1/2 and JNK emerged as the key downstream mediators of Chemerin-induced signaling in cSCC cells. This finding indicates that Chemerin, depending on the cell type and the receptor expression can activate different subtypes of MAPK pathway. The involvement of both ERK1/2 and JNK in cell migration and invasion is not unexpected and, in fact, has been well-established in various cancers [60]. We observed that the inhibition of ERK1/2 and JNK with small molecule inhibitors abolished the migration-promoting potential of Chemerin and senescent fibroblast CM in cSCC cells. This implies that majorly Chemerin, and possibly other SASP factors, reinforce MAPK as a common signaling pathway to induce tumor-cell migration. This is a clinically relevant finding indicating that the inhibition of the MAPK pathway may prevent cSCC migration and metastasis.

Apart from Chemerin, other SASP factors among them RANTES/CCL5 contribute to cSSC cell migration. The role of several SASP factors in tumor cell migration has previously been investigated. For instance, the two well-known SASP factors, CXCL8/IL-8 and CCL2/MCP-1, increase the migration of colorectal cancer cells [61]. Also, production of a Wnt antagonist named sFRP2 released from senescent dermal fibroblasts has been reported to decrease β-catenin and microphthalmia-associated transcription factor (MITF). This in conjunction with the loss of the redox effector protein APE1, a multifunctional protein with both DNA repair and transcriptional regulatory activities, eventually contributes to tumor progression and metastasis in melanoma [62].

In summary, we here uncovered the previously unreported GPR1/CCRL2/Chemerin axis in cSCC tumor cells which - via the interrelated activation of the ERK1/2 and JNK- promotes directed migration and most likely tumor progression. Given the current demographic development, this is particularly relevant as Chemerin is up-regulated in senescent fibroblasts in the skin of elderly individuals.

Our findings with the identification of target molecules responsible for skin cancer cell migration/invasion hold substantial promise for the development of novel treatment strategies to overcome the deleterious effects of SASP in elderly patients.

## MATERIALS AND METHODS

### Cell lines and primary cells

Human cutaneous squamous cell carcinoma (cSCC) lines SCC-12B2 and SCC-13 [63] were kindly provided by Dr. J.G. Rheinwald (Harvard Medical School, Boston, MA). Human SCC line A431 was purchased from the American Type Culture Collection (ATCC, Manassas, VA, USA).

Human SCL-1 cell line [64] was a gift from Dr. P. Boukamp (DKFZ, Heidelberg, Germany). Primary human dermal fibroblast (HDF) strains FF95, FFRa and FFPia, and primary normal human epidermal keratinocytes (NHEKs) were established from the foreskin of healthy males having undergone circumcision according to the method described earlier [65, 66]. Primary cSCC-associated stromal fibroblasts were derived from surplus material removed from patient biopsies (F-SCC3, 88 year-old male and F-SCC1160524, 92 year-old male) at the University Hospital Zurich, funded by the University Research Priority Program (URPP). All patients included in this study have signed a patient release form, which has been approved by an ethics committee and assigned the numbers EK647, and EK800.

### Cell culture

SCL-1, A431, HDFs and cSCC-associated primary stromal fibroblasts (F-SCC3 and F-SCC1160524) were maintained in Dulbecco's Modified Eagle's Medium (DMEM; GIBCO-BRL, Germany) supplemented with 10% fetal bovine serum (FBS; Biochrom, Berlin, Germany), 2 mM L-glutamine (Biochrom), 100 U/ml streptomycin and penicillin (Biochrom), which will be referred to as “DMEM growth media” throughout the manuscript.

NHEKs, SCC12-B2 and SCC13 cells were cultured in complete Keratinocyte Growth Medium M2 (#20011; Promocell, Heidelberg, Germany). Collagen-coated (3 μg/cm^2^) flasks were used to support the growth of Keratinocytes and SCC12B2 cells. All cells were cultured in a humidified atmosphere with 5% CO2 and 21% O2 at 37°C.

### Conditioned media

Human derma fibroblast cell strains FF95, FFRa and FFPia were passaged to reach their replicative senescence at cumulative population doublings (CPDs) > 52, 48 and 42, respectively, when the typical senescence-associated characteristics [67] appeared. To prepare the conditioned medium (CM), replicative senescent and young (CPD < 22) fibroblasts and intrinsically aged cSCC-associated primary stromal fibroblasts derived from elderly patients were seeded at a density of 1.25 × 10^4^ cells/cm^2^ in DMEM growth media. Conditioned media (CM) were harvested from confluent cells after 7 days, centrifuged (5 min, 400 × g), filtered (0.22 mm) and normalized to 5 × 10^4^ cells/ml. Aliquots were stored at −80°C until further use.

### Human skin samples

Human skin specimens were collected from old (76 ± 10) and young (21 ± 8) healthy individuals and cSCC patients (Supplementary Table S1) from the Department of Dermatology and Allergic Diseases upon approval by the Ulm University ethical committee (assigned number 155/2012) according to the Declaration of Helsinki principles, after informed written consent was obtained.

### Cell migration and invasion assays

Transwell^®^ migration assay was performed as earlier described [33] with few modifications using Corning^®^ inserts (#3422, Corning, NY, USA). Recombinant human (rh) Chemerin (#2324-CM/CF) and rh RANTES/CCL5 (#278-RN/CF) were purchased from R&D Systems (Minneapolis, MN, USA). FR180204 (#SML0320) and SP600125 (#S5567) were obtained from Sigma-Aldrich (St. Luis, MO, USA), and BIRB796 (#SM11) from Cell Guidance Systems (St. Luis, MO, USA). Stock solutions were prepared at a concentration of 60 mM in Dimethyl sulfoxide (DMSO).

In brief, a number of 2×10^5^ cells in 200 μl serum-free DMEM was placed onto the upper chambers. The lower chambers were loaded with 600 μl conditioned medium (CM) or chemotaxis factors and incubated overnight (14 h) at 37°C and 5% CO2. DMEM containing 10% fetal bovine serum (FBS) and collagen type I at a concentration of 50 μg/ml served as positive chemotaxis controls. DMEM served as a negative control. After 14 hours incubation, cells were fixed and stained using Diff Quik^®^ Stain kit (Medion Diagnostics AG, Switzerland). Non-migrated cells were removed from the upper chambers using cotton swaps. Perforated filters were cut and stuck on slides. Average number of migrated cells were counted in five different high-power microscopic fields (HPF) (×100 magnification).

Invasion assay was performed similar to the migration assay, with a difference that a layer of BD Matrigel™ (#354230, BD Biosciences, USA) was coated (100 μl/well) onto the upper filter of Transwell^®^ chambers 4 hours prior to experiment.

### Quantitative real-time PCR (qRT-PCR)

Total RNA was extracted and reverse transcribed using standard protocols. Human Chemokine and Chemokine receptor RT² Profiler™ PCR Array and specific QuantiTect primers (Qiagen, Hilden, Germany,) for RARRES2, CCRL2; GPR1, CMKLR1, CCR1, CCR2, CCR3, CCR4, CCR5, CXCR1 and CXCR2 genes were purchased from Qiagen (Supplementary Table S2) and used according to the manufacturer's instructions.

### Immunofluorescence staining

Immunofluorescence staining was performed using the previously described standard method [68]. Primary antibodies targeting the following proteins were used at indicated concentrations: Chemerin (#ab72965, 1:50, Abcam, Cambridge, UK), FSP-1 (#GTX62977, 1:200; GenetTex, Irvine, CA, USA), Cytokeratin (#M3515, 1:100, Dako, Bollschweil, Germany), CCRL2 (#ab136057, 1:100, Abcam). Appropriate isotype controls were used to determine the background. The images were acquired using AxioVision software (Carl Zeiss Microimaging GmbH, Jena, Germany).

### Western blotting

Western blot analysis was performed as earlier described [68, 69] using antibodies against CCRL2 (#ab136057, 1:1000, Abcam), total and phosphorylated MAPK member proteins (Cell Signaling, Massachusetts, USA) including p42/44 (#4695, 1:2000 & #4370, 1:2000), SEK1/MKK4 (#3346, 1:1000 & #4514, 1:1000), ATF-2 (#9226, 1:1000 & #5112, 1:1000), c-Jun (#9165 & #2361, 1:1000) and p38 (#4511, 1:2000 & #8690, 1:2000).

### Flow cytometry

CCRL2, GPR1 and CMKLR1 cell surface expression was measured using APC-conjugated anti-CCRL2 (#FAB23501A, 1:100, R&D Systems), Alexa488-conjugated anti-GPR1 mAb (#bs-13509R-A488, 1:100, Bioss, Woburn, MA, USA) and PE-conjugated CMKLR1 (#FAB362P, 1:100, R&D systems) according to the manufacturer's recommendations. Appropriate isotype controls were used to determine the background. Dead cells were excluded by co-staining with SYTOX^®^ Blue (Invitrogen, Carlsbad, CA, USA). Flow cytometry was performed on FACSCanto II (BD Biosciences) and the data were analyzed using FlowJo analysis software (TreeStar Inc.).

### ELISA

ELISAs for human Chemerin were performed using Quantikine ELISA kit (R&D Systems) according to the manufacturer's instructions.

### Cignal^™^ finder GPCR signaling reporter array

The GPCR array (#CCA-109L-2, Qiagen) was used according to manufacturer's protocol. Briefly, SCL-1 cells were seeded at a density of 4 × 10^4^ cells/well and incubated for 18 h at 5% CO_2_ and 37°C. Following transfection, medium was changed to DMEM containing 40 nM rh Chemerin. After 1h (and 24 h) incubations, cells were lysed and luciferase expression was determined using Dual-Glo^®^ Luciferase Assay according to manufacturer's instructions (Promega, Madison, WI, USA).

### SiRNA-based knockdown of Chemerin

Fibroblasts (1 × 10^5^ cells/well) were transfected with four different silencing RNAs (siRNAs; 30 nM) against Chemerin (# GS5919, Qiagen, Supplementary Table S3) or scrambled control (#1027280, Qiagen) using Lipofectamine RNAi Max Transfection Reagent (#13778, Thermo Fisher Scientific, USA). After 42 h and 48 h post transfection, quantitative RNA transcripts and secreted Chemerin in the supernatants were analyzed, respectively.

### ShRNA-mediated knockdown of CCRL2 and GPR1 in SCL-1

For the stable knockdown of CCRL2 and GPR1, short hairpin RNA (shRNA) plasmids were obtained from Genecoepia (Rockville, MD, USA, Supplementary Table S4). HEK293T cells (4 × 10^6^ /10cm dish) were transfected with 13 μg shRNA-plasmids along with 8 μg psPAX2 and 4 μg pMD2 packaging plasmids (Addgene, Cambridge, MA, USA) using TransIT-LTR transfection reagent (Mirus Bio LLC, Madison, WI, USA), and following manufacturer's instructions. Virus was collected 72 hours after transfection, filtered, and stored at 4°C for a maximum of one day. The virus titer was assessed and normalized based on the viral protein P24 measurements using a P24 ELISA kit (XpressBio, USA). A number of 2 × 10^6^ SCL-1 cells were transduced using equal virus titer. Transduction efficiency was monitored based on eGFP/mCherry expression, and positively-transduced SCL-1 cells were sorted using FACSAria III (BD Biosciences). Knock-down efficacy was measured using qRT-PCR analysis.

### MTT cell viability/proliferation assay

Cell Proliferation Kit I (MTT) (Roche applied science; Indianapolis, IN) was used for the quantitation of living metabolically active cells as previously described [70]. In brief, SCL-1 cells (1 × 10^4^ cells/well) were seeded in 96-well plates and serum-starved on the next day. After 24 hours, media was exchanged with DMEM containing rh Chemerin (or DMEM growth media containing MAPK inhibitors) and incubated for 14, 24, 36 and 48 hours.

### BrdU proliferation assay

The effect of Chemerin on the proliferation of SCL-1 was examined by measuring BrdU incorporation into newly-synthesized DNA using immune fluorescence microscopy and flow cytometric analysis as described earlier [70].

### Statistics

The final results were calculated as mean ± standard error (SEM) of three independent experiments. In some experiments, one of three independent experiment was presented as mean ± standard deviation (SD). Two-tailed unpaired student *t*-test and on-way ANOVA with Bonferroni post hoc test were performed as appropriate, with significance at *p* ≤ 0.05 using GraphPad Prism Software Inc (London, UK) unless otherwise stated.

## SUPPLEMENTARY MATERIALS



## Abbreviations

ANOVA: Analysis of Variance
BrdU: 5-bromo-2′-deoxyuridine
cSCC: Cutaneous squamous cell carcinoma
CCRL2: Chemokine CC-motif receptor-like 2
CM: Conditioned medium
CMKLR1: Chemokine-like receptor 1
DMEM: Dulbecco's Modified Eagle's Medium
DMSO: Dimethyl sulfoxide
ELISA: Enzyme-linked immunosorbent assay
ERK: Extracellular-signal-regulated kinase
FBS: Fetal bovine serum
GFP: Green fluorescent protein
GPCRs: G-Protein coupled receptors
GPR1: G-protein coupled receptor 1
HDF: Human dermal fibroblast
JNK: c-Jun N-terminal kinase
MAPK: Mitogen-activated protein kinase
NHEKs: Normal human epidermal keratinocytes
qRT-PCR: quantitative reverse transcription-polymerase chain reaction
RANTES: Regulated on activation, normal T cell expressed and secreted
RARRES2: Retinoic acid receptor responder 2
SASP: Senescence-associated secretory phenotype
SD: Standard derivation
SEM: Standard error of the Mean
ShRNA: Short hairpin RNA
SiRNA: Small interfering RNA
TIG2: Tazarotene-induced gene 2
