# Supplementary material for: Senescent fibroblast-derived Chemerin promotes squamous cell carcinoma migration
Source: Oncotarget. 2016 Nov 18;7(50):83554–69. doi: 10.18632/oncotarget.13446 (PMC5347788; doi:10.18632/oncotarget.13446)
Supplement: Supplementary file 1 [file oncotarget-07-83554-s001.pdf]

# Senescent fibroblast-derived Chemerin promotes squamous cell carcinoma migration

## Supplementary Materials

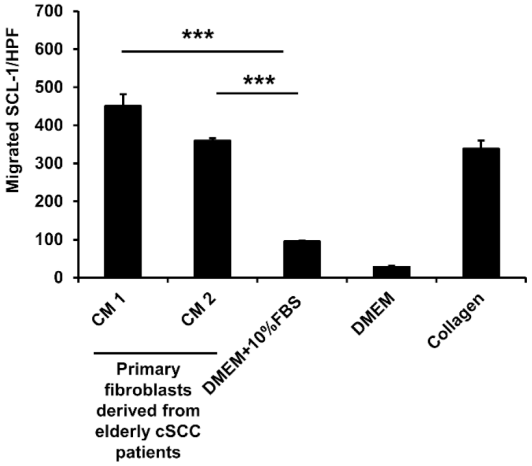


**Supplementary Figure S1: Migration of SCL-1 cells in response to conditioned media derived from intrinsically aged primary fibroblast of cSCC patients.** Conditioned media (CM) was prepared from two different cSCC-associated stromal fibroblasts from elderly cSCC patients at an age of 88 years and 92 years (F-SCC3 and F-SCC1160524) to induce the migration of SCL-1 cells using the Transwell® chamber migration assay. DMEM containing 10% FBS and collagen type I at a concentration of 50 µg/ml served as positive controls. DMEM served as a negative control. Displayed are the result of migration assay using two different passages of primary fibroblast F-SCC1160524 (labeled as CM1 and CM2). The results indicate that CM of primary fibroblasts of elderly cSCC patients significantly enhance the migration of SCL-1 compared to DMEM containing 10% FBS. Data are shown as mean ± S.D with $n = 3$ replicate wells; ***$p < 0.001$ calculated by unpaired student *t*-test between CM-treated and DMEM-10% FBS treated groups; HPF= ×100 magnification. Similar results were obtained using primary aged stromal fibroblast F-SCC3 (CM1 = 351 ± 43 cells/HPF and CM2 = 333 ± 52 cells/HPF).

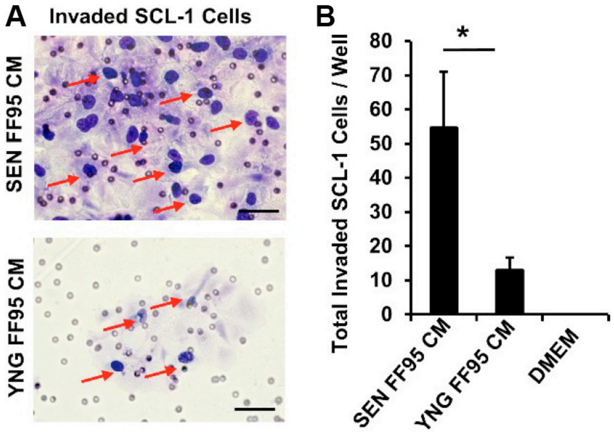


**Supplementary Figure S2: Secretome of senescent fibroblasts enhances the invasion of SCL-1 tumor cells.** Cell invasion assay was performed similar to migration assay apart from coating a layer of BD Matrigel™ was coated (100 µl/well) onto the upper filter of Transwell® chambers 4 hours prior to the experiment. The lower compartments of the chambers were loaded with CM of senescent and young FF95 fibroblasts and incubated for 14 h at 37°C. DMEM served as a control to monitor random invasion. Cells stained purple by Diff-Quick staining kit and counted under light microscopy at ×100 magnification (**A**) Representative images (×200 magnification, scale bars = 50 µm) showing invaded SCL-1 cells (pointed with red arrows) at the downside of the membrane in response to CM from senescent vs. young fibroblasts. (**B**) Quantifications of cell invasion proved that senescent FF95 CM increased the invasion of SCL-1 tumor cells. Graph is a representative of three independent experiment. *$p < 0.05$ calculated by unpaired student *t*-test; HPF= ×100 magnification.

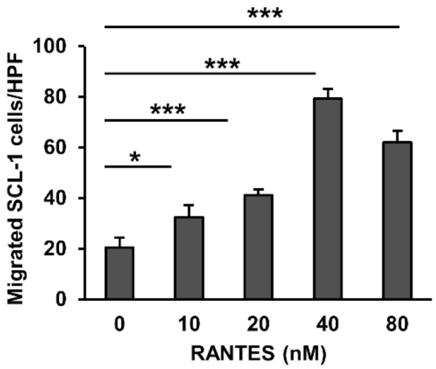


**Supplementary Figure S3: Effect of rh RANTES/CCL5 chemokine on SCL-1 cell migration.** Effect of rh RANTES/CCL5 chemokine on SCL-1 cell migration. The graph depicts the quantification of migrated SCL-1 cells after 14 h treatment with increased concentration of rh RANTES using Transwell chamber migration assay. Results are expressed as the mean number of migrated cells ± S.D for $n = 4$ replicate wells (HPF = ×100 magnification). The results are representative of one of three independent experiments. *$p < 0.05$, **$p < 0.01$ and ***$p < 0.001$ calculated by Bonferroni post hoc test after ANOVA.

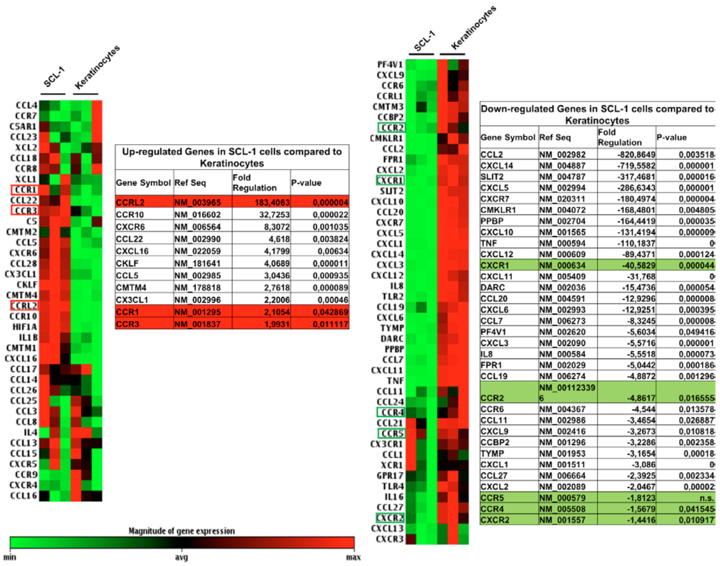


| Up-regulated Genes in SCL-1 cells compared to Keratinocytes | | | |
|---|---|---|---|
| Gene Symbol | Ref Seq | Fold Regulation | P-value |
| CCRL2 | NM_003965 | 183,4063 | 0,000004 |
| CCR10 | NM_016602 | 32,7253 | 0,000022 |
| CXCR6 | NM_006564 | 8,3072 | 0,001035 |
| CCL22 | NM_002990 | 4,618 | 0,003824 |
| CXCL16 | NM_022059 | 4,1799 | 0,00634 |
| CKLF | NM_181641 | 4,0689 | 0,000011 |
| CCL5 | NM_002985 | 3,0436 | 0,000935 |
| CMTM4 | NM_178818 | 2,7618 | 0,000089 |
| CX3CL1 | NM_002996 | 2,2006 | 0,00046 |
| CCR1 | NM_001295 | 2,1054 | 0,042869 |
| CCR3 | NM_001837 | 1,9931 | 0,011117 |

| Down-regulated Genes in SCL-1 cells compared to Keratinocytes | | | |
|---|---|---|---|
| Gene Symbol | Ref Seq | Fold Regulation | P-value |
| CCL2 | NM_002982 | -820,8649 | 0,003518 |
| CXCL14 | NM_004887 | -719,5582 | 0,000001 |
| SLIT2 | NM_004787 | -317,4681 | 0,000016 |
| CXCL5 | NM_002994 | -286,6343 | 0,000001 |
| CXCR7 | NM_020311 | -180,4974 | 0,000004 |
| CMKLR1 | NM_004072 | -168,4801 | 0,004805 |
| PPBP | NM_002704 | -164,4419 | 0,000035 |
| CXCL10 | NM_001565 | -131,4194 | 0,000009 |
| TNF | NM_000594 | -110,1837 | 0 |
| CXCL12 | NM_000609 | -89,4371 | 0,000124 |
| CXCR1 | NM_000634 | -40,5829 | 0,000044 |
| CXCL11 | NM_005409 | -31,768 | 0 |
| DARC | NM_002036 | -15,4736 | 0,000054 |
| CCL20 | NM_004591 | -12,9296 | 0,000008 |
| CXCL6 | NM_002993 | -12,9251 | 0,000395 |
| CCL7 | NM_006273 | -8,3245 | 0,000008 |
| PF4V1 | NM_002620 | -5,6034 | 0,049416 |
| CXCL3 | NM_002090 | -5,5716 | 0,000001 |
| IL8 | NM_000584 | -5,5518 | 0,000073 |
| FPR1 | NM_002029 | -5,0442 | 0,000186 |
| CCL19 | NM_006274 | -4,8872 | 0,001296 |
| CCR2 | NM_001123396 | -4,8617 | 0,016555 |
| CCR6 | NM_004367 | -4,544 | 0,013578 |
| CCL11 | NM_002986 | -3,4654 | 0,026887 |
| CXCL9 | NM_002416 | -3,2673 | 0,010818 |
| CCBP2 | NM_001296 | -3,2286 | 0,002358 |
| TYMP | NM_001953 | -3,1654 | 0,00018 |
| CXCL1 | NM_001511 | -3,086 | 0 |
| CCL27 | NM_006664 | -2,3925 | 0,002334 |
| CXCL2 | NM_002089 | -2,0467 | 0,00002 |
| CCR5 | NM_000579 | -1,8123 | n.s. |
| CCR4 | NM_005508 | -1,5679 | 0,041545 |
| CXCR2 | NM_001557 | -1,4416 | 0,010917 |

**Supplementary Figure S4: Expression profile of chemokine and chemokine receptors in SCL-1 cell line compared to normal keratinocytes.** Heat maps illustrate differentially regulated chemokine and chemokine receptor genes in SCL-1 cell line compared with normal keratinocytes. Red and green colors represent upregulation and down-regulation, respectively. Tables indicate fold changes and significance levels in mRNA expression of chemokines and their receptors in SCL-1 cell line compared to normal keratinocytes. The expression level of selected chemokine receptors (shown with boxes), which were relevant to SASP-induced migration, was further assessed using qRT-PCR analysis. Of note, CCRL2 has the highest fold regulation among other chemokine receptors expressed in SCL-1 tumor cells.

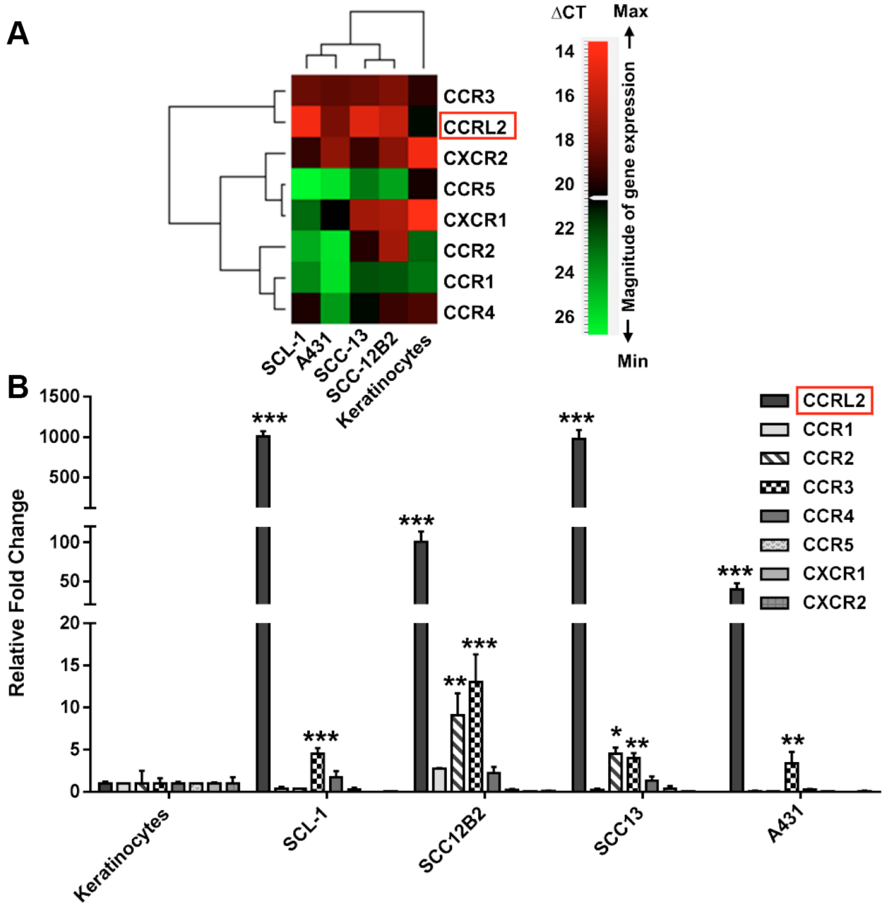


**Supplementary Figure S5: Expression pattern of selected chemokine receptors in cSCC cell lines vs. normal keratinocytes.** Quantitative RT–PCR for selected chemokine receptors was performed to validate the RT2 Profiler PCR data in keratinocytes vs. cSCC cell lines (SCL-1, SCC12B2, SCC-13 and A431) (**A**) Heat map illustrating differentially expressed chemokine receptors. Signal intensities were averaged between biological replicates of three independent experiments and normalized to the averaged signal from biological replicates of normal keratinocytes. Green indicates downregulation (lower delta Ct values) and red upregulation (higher delta Ct values). (**B**) Graph represents the relative fold changes in mRNA expression normalized to the average expression level in keratinocytes. The qRT–PCR experiment was performed independently three times using samples prepared separately from the PCR array experiment. Data are shown as mean ± S.D for one of three independent experiments with $n = 3$; *$p < 0.05$, **$p < 0.01$ and ***$p < 0.001$ calculated by Bonferroni post hoc test after ANOVA shown for upregulated genes in cSCC cell lines as compared to normal keratinocytes.

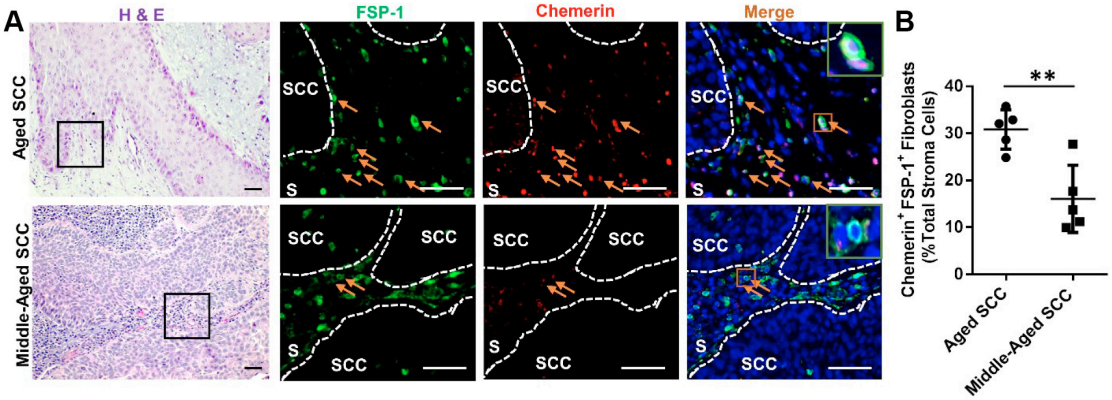


**Supplementary Figure S6: Immunofluorescence staining of Chemerin and the fibroblast marker FSP-1 in paraffin-embedded skin sections of cSCC patient biopsies.** (**A**) Representative microphotographs of skin sections derived from an aged cSCC patient (83 years), and relatively younger patient (61 years), stained for the fibroblast marker FSP-1 (green), Chemerin (red), and with DAPI for nuclei (blue). Scale bars = 50 µm at × 400 magnification; Dashed lines delineate SCC from stroma (S). Orange arrows point to the Chemerin positive fibroblasts, indicating higher numbers of Chemerin expressing fibroblasts in sections from a very old cSCC patient compared to middle-aged cSCC patient. (**B**) Graph representing the quantification of percentage of FSP-1 and Chemerin double-positive cells normalized to the total number of nuclei in the tumor stroma of cSCC patients at different age groups (80 ± 5 years, $n = 5$ cSCC patients) and middle-aged (54 ± 5 years, $n = 5$ donors). Unpaired student t-test revealed statistical significant differences (**$p < 0.01$) between the two groups.

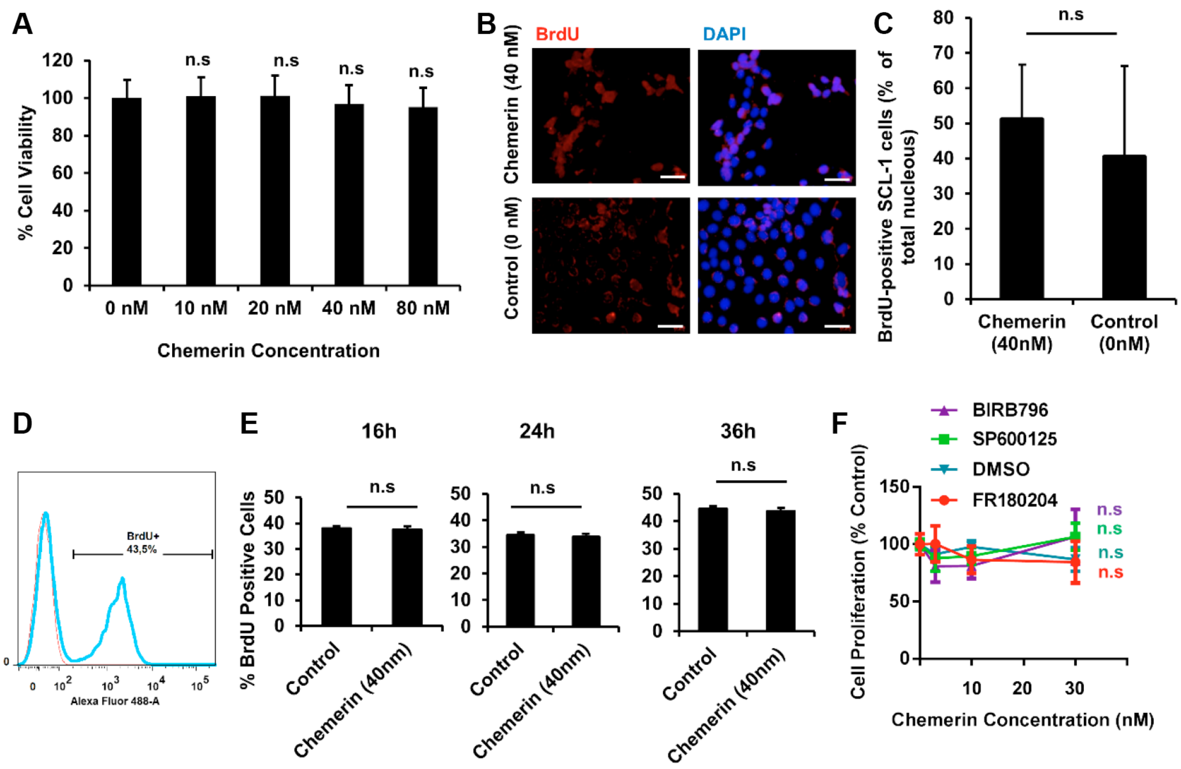


**Supplementary Figure S7: SCL-1 cell proliferation/survival analysis in response to rh Chemerin and MAPK inhibitors using MTT and BrdU assays.** (**A**) Graph demonstrating that Chemerin has no significant effect on the percentage of metabolically active living cells as defined by MTT assay, indicating that Chemerin probably does not induce pathways involved in cell proliferation, viability and metabolism. Data are shown as the mean percentage ± S.E.M live metabolically active SCL-1 cells treated with Chemerin compared to untreated cells (*n* = 5 independent experiments; n.s. = non. Significant as indicated with ANOVA). (**B**–**E**) To assess the effect of Chemerin on cell proliferation, SCL-1 cells were cultured with (or without) Chemerin for the indicated times, pulse labeled with BrdU for 2 hours, harvested and fixed. DNA synthesis was assessed by staining with an antibody to BrdU, and measured by (B and C) immunofluorescence microscopy, and (D and E) flow cytometry analysis. (B) Representative pictures of immunofluorescence staining showing the expression of BrdU-incorporated SCL-1 cells after 24 hour treatment with 40 nM Chemerin compared with untreated controls. Cells were stained with anti- BrdU specific mAb in red. Nuclei were DAPI-counterstained in blue. Scale bars = 50 µm. (**C**) Quantification of BrdU-incorporated SCL-1 nuclei in Chemerin-treated and untreated SCL-1 cells. Results are the mean ± S.D. of three biological replicates. n.s.= non-significant as calculated with unpaired student *t*-test. (D) Histogram representative of flow cytometric analysis showing the percentage of BrdU-positive SCL-1 cells as defined by mean fluorescent intensity of anti-BrdU antibody conjugated with Alexa-fluor488. The solid blue line depicts BrdU expression and red histogram is negative control with no DNA-incorporation of BrdU. (E) Bar charts represent one of three independent experiments demonstrating the mean percentage ± S.D. of BrdU incorporated SCL-1 cells, showing no statistically significant difference as analyzed by unpaired student *t*-test. (**F**) Graph demonstrating the cytotoxicity of MAPK inhibitors on SCL-1 cells as defined by MTT assay. Cytotoxicity was calculated as the percentage of metabolically active living cells treated with different agents compared with cells treated with DMSO as a vehicle control. None of the inhibitors has cytotoxic effect on SCL-1 cells as computed by linear regression analysis. Data points are mean ± S.D for five samples per MAPK inhibitor. The experiment was repeated five times. $p < 0.05$, $**p < 0.01$ and $***p < 0.001$.

## Supplementary Table S1: List of cSCC and normal skin samples used for immunostaining

| Sample Nr. | Tissue type/ Diagnisis | Gender | Age | Location | Broder's classification |
|---|---|---|---|---|---|
| 1 | cSCC | F | 90 | Hairy head skin | Moderately-differentiated |
| 2 | cSCC | F | 87 | Cervical spine | Well-differentiated |
| 3 | cSCC | M | 85 | Temple | Moderately-differentiated |
| 4 | cSCC | M | 83 | Lower lip | Moderately-differentiated |
| 5 | cSCC | M | 83 | Forehead | Moderately-differentiated |
| 6 | cSCC | M | 83 | Hand | Moderately-differentiated |
| 7 | cSCC | F | 75 | Nose | Moderately-differentiated |
| 8 | cSCC | M | 75 | Nose | Poorly-differentiated |
| 9 | cSCC | M | 74 | Lower arm | Well-differentiated |
| 10 | cSCC | M | 73 | Hairy head skin | Well-differentiated |
| 11 | cSCC | F | 72 | Forehead | Well-differentiated |
| 12 | cSCC | F | 61 | Nose | Moderately-differentiated |
| 13 | cSCC | F | 56 | Cheek | Well-differentiated |
| 14 | cSCC | M | 52 | Cheek | Moderately-differentiated |
| 15 | cSCC | M | 50 | In front of ear | Well-differentiated |
| 16 | cSCC | M | 50 | In front of ear | Moderately-differentiated |
| 17 | Normal Skin | F | 91 | Back | |
| 18 | Normal Skin | M | 89 | Left occipital | |
| 19 | Normal Skin | M | 82 | Back | |
| 20 | Normal Skin | M | 82 | Sternal | |
| 21 | Normal Skin | M | 79 | Back | |
| 22 | Normal Skin | M | 79 | Left shoulder | |
| 23 | Normal Skin | M | 77 | Back | |
| 24 | Normal Skin | F | 74 | Right neck | |
| 25 | Normal Skin | F | 74 | Lower arm | |
| 26 | Normal Skin | M | 73 | Back | |
| 27 | Normal Skin | F | 72 | Left shoulder | |
| 28 | Normal Skin | F | 72 | Back | |
| 29 | Normal Skin | M | 71 | Ear | |
| 30 | Normal Skin | M | 70 | Back | |
| 31 | Normal Skin | M | 47 | Back | |
| 32 | Normal Skin | M | 42 | Back | |
| 33 | Normal Skin | M | 36 | Hairy head skin | |
| 34 | Normal Skin | F | 31 | Knee | |
| 35 | Normal Skin | F | 24 | Back | |
| 36 | Normal Skin | F | 24 | Back | |
| 37 | Normal Skin | M | 23 | Schoulder | |
| 38 | Normal Skin | M | 19 | Lower left thigh | |
| 39 | Normal Skin | F | 18 | Back | |
| 40 | Normal Skin | F | 16 | Armpit | |
| 41 | Normal Skin | M | 16 | Left paravertebral | |
| 42 | Normal Skin | M | 16 | Back | |
| 43 | Normal Skin | F | 16 | Buttocks | |
| 44 | Normal Skin | F | 16 | Breast skin | |
| 45 | Normal Skin | F | 15 | Upper stomach skin | |
| 46 | Normal Skin | F | 15 | Forhead | |
| 47 | Normal Skin | F | 13 | Back | |
| 48 | Normal Skin | M | 13 | Left foot sole | |
| 49 | Normal Skin | F | 9 | Back | |
| 50 | Normal Skin | M | 5 | Back | |

**Supplementary Table S2: List of primers used for qRT-PCR purchased from qiagen (hilden, germany)**

| Gene | Product Name | CAT No. C |
|---|---|---|
| Actin | QuantiTect Primer Assay Hs_ACTB_2_SG | QT01680476 |
| CCL5 | QuantiTect Primer Assay Hs_CCL5_1_SG | QT00090083 |
| CCR1 | QuantiTect Primer Assay Hs_CCR1_1_SG | QT00047740 |
| CCR2 | QuantiTect Primer Assay Hs_CCR2_1_SG | QT00000224 |
| CCR3 | QuantiTect Primer Assay Hs_CCR3_2_SG | QT02423596 |
| CCR4 | QuantiTect Primer Assay Hs_CCR4_1_SG | QT00208950 |
| CCR5 | QuantiTect Primer Assay Hs_CCR5_1_SG | QT00998802 |
| CCRL2 | QuantiTect Primer Assay Hs_CCRL2_1_SG | QT00225988 |
| CMKLR1 va. 1 | QuantiTect Primer Assay Hs_CMKLR1_va.1_SG | QT02394707 |
| CMKLR1 va. 2 | QuantiTect Primer Assay Hs_CMKLR1_2_SG | QT01680140 |
| CXCR1 | QuantiTect Primer Assay Hs_CXCR1_1_SG | QT00212919 |
| CXCR2 | QuantiTect Primer Assay Hs_CXCR2_1_SG | QT00000518 |
| GPR1 | QuantiTect Primer Assay Hs_GPR1_1_SG | QT00209748 |
| RARRES2 | QuantiTect Primer Assay Hs_RARRES2_1_SG | QT00091945 |

**Supplementary Table S3: List of siRNAs used for the knockdown of Chemerin (RARRES 2 gene) purchased from qiagen company**

| Label | CAT No. | Target Sequence |
|---|---|---|
| RARRES2 siRNA 1 | SI00045318 | CTGGAAGAAACCCGAGTGCAA |
| RARRES2 siRNA 2 | SI00045325 | CCCAATGGGAGGAAACGGAAA |
| RARRES2 siRNA 5 | SI03094469 | CTGCATCAAACTGGGCTCTGA |
| RARRES2 siRNA 6 | SI03102127 | GACGGCCAGGGTGACACGGAA |
| Scrambled Control | 1027280 | (AllStars Negative Control) |

**Supplementary Table S4: List of shRNAs used for CCRL2 and GPR1 receptor knockdown in SCL-1 cells purchased from Genecoepia (Rockville, MD, USA)**

| Label | Product Name | Target Gene | Accession No. | Reporter Gene | Target Sequence |
|---|---|---|---|---|---|
| CCRL2 shRNA 1 | HSH021922-1-LVRH1GP(CS9OS397048) | CCRL2 | NM_003965.4 | eGFP | GCAGAGCAATGTGACAAGTAT |
| CCRL2 shRNA 2 | HSH021922-2-LVRH1GP(CS9OS397049) | CCRL2 | NM_003965.4 | eGFP | GCATCATTACAAGTGTCCTGG |
| CCRL2 shRNA 3 | HSH021922-3-LVRH1GP(CS9OS397050) | CCRL2 | NM_003965.4 | eGFP | GACCAGAAATACAAGTGTGCA |
| CCRL2 shRNA 4 | HSH021922-4-LVRH1GP(CS9OS397051) | CCRL2 | NM_003965.4 | eGFP | GCAAATACCTCTGCCGCTGTT |
| CCRL2 Scrambled Control | CSHCTR001-1-LVRH1GP(OSNEG20) | - | - | eGFP | GCTTCGCGCCGTAGTCTTA |
| GPR1 shRNA 5 | HSH007828-5-LVRH1MP(OS397976) | GPR1 | NM_005279.3 | mCherry | GCTACTTGTGTCTCATCTTCA |
| GPR1 shRNA 5 | HSH007828-6-LVRH1MP(OS397977) | GPR1 | NM_005279.3 | mCherry | GCTGGACTCCTTATCACCTGT |
| GPR1 shRNA 5 | HSH007828-7-LVRH1MP(OS397978) | GPR1 | NM_005279.3 | mCherry | GCTCACCATTCACCACAATAG |
| GPR1 shRNA 5 | HSH007828-8-LVRH1MP(OS397979) | GPR1 | NM_005279.3 | mCherry | GGTCCTCAGTTGCTGAGATAC |
| GPR1 Scrambled Control | CSHCTR001-1-LVRH1MP(OSNEG20) | - | - | mCherry | GCTTCGCGCCGTAGTCTTA |
